# Characterizing Organizational Lifecycle through Strategic and Structural Flexibility: Insights from MSMEs in Mexico

**DOI:** 10.1007/s40171-022-00301-4

**Published:** 2022-03-21

**Authors:** Adrianela Angeles, Adriana Perez-Encinas, Cristian E. Villanueva

**Affiliations:** 1 Facultad de Economía Y Negocios, Universidad Anáhuac, Huixquilucan, Edo. de México México; 2 Business and Economics School, Universidad Autónoma de Madrid, Madrid, Spain

**Keywords:** Decentralized decision making, Decision tree analysis, Developing country, Organizational growth, Proactive flexibility, Two-step cluster analysis

## Abstract

Today’s lifespan of companies tends to be low in the so-called micro-, small-, and medium-sized enterprises (MSMEs). Organizational life cycle (OLC) theory indicates that organizational aging is related, but not determined, by the firm chronological age or its size. Therefore, a firm’s aging should be analyzed by other factors such as flexibility. The literature considers flexibility as an essential capability, a source of competitive advantage, and an enabler of long-term growth for MSMEs. However, little attention in emerging economies has been paid to examine the nuances of this concept in relation to the OLC in this type of companies. Additionally, studies tend to analyze flexibility as a general term, ignoring that it is a polymorphic concept. That is why there is a need to research the different categories of flexibility. Drawing on a quantitative approach conducting a factor analysis, a two-step cluster, and decision tree analysis to interrogate data from 257 MSMEs in Mexico, this study provides evidence of different dimensions of strategic and structural flexibility that help to characterize and predict the growth, maturity, and declining stages of MSMEs. Our results show that mature firms present more strategic and structural flexible characteristics than those involved in growth or decline stages. The flexible factors that help classify and predict an MSME in the maturity stage include open communication, decentralized decision making, and formalization. We provide a model with these results to illuminate unaddressed issues regarding the broad term of flexibility and its relationship to OLC.

## Introduction

The current business scenario faces several global challenges, such as environmental crises, technological disruption (Bishwas & Sushil, 2020), and more recently, the global and persistent Covid-19 pandemic (Masudin et al., 2021; Mokline & Ben Abdallah, 2022). All these circumstances have exposed the shortcomings of the traditional managerial approaches (Majid et al., 2019; Settembre-Blundo et al., 2021) and have prompted an increase in flexibility research, a term that has emerged as a linking concept that allows organizational agility to address such uncertainties and threats (Evans & Bahrami, 2020; Momaya et al., 2017; Subramanian & Suresh, 2022).

Flexibility is consistently associated with micro-, small-, and medium-sized enterprises (MSMEs) as one of their competitive advantages (Zhang et al., 2014), an important facilitator of their long-term growth (Celuch & Murphy, 2010) and a key strategy for the survival of local businesses when competing with large firms (Momaya et al., 2017). The MSME sector is the backbone of world economies and makes outstanding contributions to employment and added value added for developed and developing countries (OECD, 2017). However, statistics show a high mortality rate among them. For instance, according to the U.S. Small Business Administration (2019), from 1994 to 2018, 67.6% of small businesses survived at least two years. The five-year survival rate was 48.8%; at ten years, it was 33.6%; and at fifteen years it was only 25.7%. Furthermore, the failure rate of MSMEs may have increased 9.1 percent due to the impact of COVID-19 (Gourinchas et al., 2020).

Organizational life cycle (OLC) theory emphasizes that organizations evolve independently from their size and chronological age, and is the decrease in flexibility and adaptability which determine youth or organizational aging (Adizes, 1979; Mosca et al., 2021). This leads us to question what particular flexibility factors can help to characterize and predict the growing, mature and declining OLC stages in MSMEs. Today’s organizations demand more comprehensive and integrated approaches in this regard that allow them to respond promptly to external changes (Majid et al., 2019; Settembre-Blundo et al., 2021) and avoid failure, this is crucial especially for MSMEs in transitional countries (Milošević et al., 2019).

Several studies have been made efforts to comprehend flexibility as a source of competitive advantage (Dubey et al., 2021; Wadhwa & Rao, 2004), driver of innovation (Miroshnychenko et al., 2020), and for superior supply chain performance (Singh et al., 2021). Nevertheless, its relationship to the OLC has been relatively neglected. Furthermore, some flexibility studies have analyzed this term mainly as a general, abstract, and homogeneous concept (Dubey et al., 2021; Sharma et al., 2010; Zhang et al., 2014), overlooking that it is a polymorphous and context-specific construct (Evans & Bahrami, 2020).

Additionally, most of these studies have been carried out in developed countries (Zhou & Wu, 2010), where organizational survival rates tend to be higher than those of their counterparts of emerging economies. For instance, in a bibliometric analysis from a top journal in flexibility “Global Journal of Flexible Systems Management” carried out in the last two decades, only 4% of total articles came from countries in South America (Singh et al., 2021). In this sense, more contextualized studies, especially in developing nations, are still needed (Carrillo, 2007) as the models from mainstream settings may not fit them (Angeles et al., 2019). In addition, calls have been put forward for more studies that analyze flexibility variants in MSMEs due to their particular formalization processes and structural characteristics (Jirásek & Bílek, 2018).

In this article, we examine the particularities of two types of flexibility that are especially important for dealing with turbulent environments: strategic and structural flexibility. The former represents the firm’s ability to respond to changes in its external environment in ways that lead to success, survival, and longevity (Brozovic, 2018; Zahra et al., 2008). The latter refers to a company’s capacity to change its structure and processes to adjust to environmental changes (Amarikwa et al., 2020). We analyze them throughout the growth, maturity, and decline OLC stages using unique survey data from 257 MSMEs.

The contribution of this study is to provide empirical evidence of what particular strategic and structural flexibility factors help to characterize and predict the growing, mature, and declining stages of the MSMEs. In this way, we extend the OLC model and join the efforts of more authors to move toward a more robust OLC theory.

The remainder of this article is structured as follows. In next section, we present the context of the research, and the literature review on OLC and stratregic and structural flexibility, followed by the methodology. Next section describes the data analysis and results. The discussion of our results will follow with a conclusion with a series of theoretical and managerial implications, as well as limitations and further research directions.

## Literature Review

Following other authors (Fredericks, 2005; Zhang et al., 2014), we used contingency theory to better understand the importance of flexibility during the firm life cycle. Contingency theory states that organizational effectiveness is the result of fitting the firm’s structure and strategy to contingencies (environmental demands) (Donaldson, 2001; Fredericks, 2005). Therefore, there is no “universal strategy” or an “ideal combination of resources” to deal with environmental dynamism since the strategies and structures are firm and context-specific. Companies that are best aligned with emerging environmental changes are better equipped to survive (Donaldson, 2001; Fredericks, 2005; Volberda, 1999). For that reason, although flexibility research contemplates different categories, such as financial flexibility, manufacturing flexibility, marketing flexibility, strategic flexibility, and supply chain flexibility (Singh et al., 2021); strategic and structural flexibility are especially relevant for this study, since they guarantee a better fit between the internal characteristics of companies and their external demands (Furr et al., 2012; Zahra et al., 2008).

### MSMEs in the Mexican Context

According to the information provided by the National Mapping Agency and Bureau of the Census (INEGI, 2019), there are 6.3 million companies in Mexico and 99.8% of them are MSMEs. They account for 67.9% of total employed personnel and contribute to 45.3% of total gross production (INEGI, 2020). In this context, large size firms are a minority in terms of business units. Table 1 shows the classification characteristics of Mexican companies by size.

**Table 1:** Characteristics of Mexican companies by size

Company by size	Employees	Yearly turnover (million pesos)	% of total national firms (%)	% of total employed personnel (%)
Micro	1–10	$4	94.9	37.2
Small	11–50	$100	4.0	14.8
Medium	51–250	$250	0.9	15.9
Large	More than 250	More than 250	0.2	32.1

Source: INEGI (2019)

In relation to the MSMEs economic sector, the vast majority are commercial companies, followed by service firms, and finally industrial companies (INEGI, 2014). Table 2 shows these percentages in terms of business units, employed personnel, and contribution to gross production. It also shows the life expectancy of these companies.

**Table 2:** Characteristics of Mexican companies by activity sector

Activity sector	Life expectancy (years)	Percentage of companies (%)	Employed personnel (%)	Gross production contribution (%)
Industry	9.7	12.1	23.9	48.2
Service	8	39.6	40.0	23.2
Commerce	6.9	46.8	27.6	13.6

Sources: INEGI (2020, 2019)

The main lines of business in the commercial sector, by company size, are: grocery (micro-businesses), fuels and lubricants (small enterprises), food wholesale (medium-sized companies), and retail stores (large firms). The industry sector in Mexico is highly diversified. The main activity of the micro-industries is the elaboration of bakery products and tortillas; small businesses are largely engaged in garment manufacturing; medium-sized companies are mainly dedicated to producing plastic articles; and large companies are predominantly auto parts manufacturers (INEGI, 2014). In the service sector, hotels and restaurants, as well as educational services are the main lines of business for micro-, small-, and medium-sized companies. Large companies are mostly dedicated to providing business support and waste management services (INEGI, 2014).

Finally, by geographic area, industrial MSMEs are located mainly in the southeast and northeast regions of the country, while the central region (Mexico City, Hidalgo, State of Mexico, Morelos, Querétaro, and Tlaxcala) concentrates the majority of MSMEs dedicated to commercial and service activities (INEGI, 2014).

When comparing the statistics of MSMEs from the last two national censuses (INEGI, ) we found that the previous data related to the economic sectors have not undergone significant changes during the last 5 years.

Mexican MSMEs are typically family owned, where the owner is the one who directs the company’s growth and transformation (Cantú et al., 2021). MSMEs operating in developing countries face various obstacles and challenges, for instance, poor strategic vision of owner–managers (Valdez-Juárez et al., 2021) and limited resources (Cantú et al., 2021). Particularly in Mexico, limited internet connectivity coverage (Valdez-Juárez et al., 2021), insecurity, high operating expenses and taxes hinder the development of MSMEs (INEGI, 2020). This highlights an issue worthy of consideration and the need for more empirical studies on MSMEs in developing economies to help them meet such environmental challenges.

### Strategic and Structural Flexibility

Strategic flexibility is one of the most relevant and difficult capabilities that managers in dynamic environments must promote and maintain (Shimizu & Hitt, 2004). It has been identified as an important enabler of long-term MSME growth (Celuch & Murphy, 2010) and a predictor of the vitality and sustainability of an enterprise (Sushil, 2011). Firms with greater strategic flexibility are capable to distinguish significant changes in organizational innovative activities and catalyze existing resources quickly to give response to environmental changes (Jia et al., 2021). For those reasons, it is not surprising that it is the main category of flexibility studied in flexible systems management research during the last twenty years (Singh et al., 2021).

Strategic flexibility can be applied at two levels: the firm level or flexible maneuver approach, and the decision-maker level or the flexible cognitive style approach (Combe & Greenley, 2004). Following the maneuver approach (level of the firm), it can be said strategic flexibility is related not only to how firms reactively respond to environmental changes, but also to how they proactively attempt to transform their context and create new opportunities (Herhausen et al., 2021). Reactive or internal strategic flexibility operates within the firm with the aim of adapting to the environment. It can be generated through the redefinition of organizational strategy, the versatility of resources, and the implementation of new technologies (Guo & Cao, 2014; Tamayo-Torres et al., 2010). Proactive or external strategic flexibility refers to a company’s ability to influence its environment to make the firm less vulnerable to changes (Tamayo-Torres et al., 2010). This broader level of flexibility implies greater abilities to change game plans, to act on opportunities, or simply to be “proactive” in addressing changes in the business (Brozovic, 2018). It can be achieved by the renewal of product–market combinations, influencing consumers’ behavior through advertising, using market power to stop/control the entrance of new competitors, or by participating in political activities to neutralize trade laws (Sharma et al.,  2010; Volberda, 1996).

However, despite the relevance of strategic flexibility, there are controversies about whether it is more a characteristic of large or small companies, and if the older ones are more capable of it than the new ones. Some studies describe strategic flexibility as a feature of large companies because of their potential availability of resources, so they can be less prone to rigidity, especially in decline stages (Barker & Barr, 2002; Pauwels & Matthyssens, 2004*).* Other scholars point out that large firms might avoid making strategic changes because they want to maintain their status quo, so they tend to have a higher structural inertia than smaller ones (Nadkarni & Herrmann, 2010). Old and well-established companies are also subject to inertial constraints that would prevent them from changing their strategy in significant ways (Ebben & Johnson, 2005).

In contrast, some research studies identify that strategic flexibility can be manifested by smaller businesses because they have greater adaptability of their resources (Ebben & Johnson, 2005). The agility derived from their small organizational structure allows them to take advantage of strategic tools (Zhang et al., 2014). In addition, it has been observed that family firms have more flexibility than their counterparts, non-family ones (Rastogi et al., 2016), due to their simpler organizational design (Broekaert et al., 2016). Additionally, some recent studies found that firm size and age do not inhibit strategic flexibility (Herhausen et al., 2021) and, although the literature may serve to frame strategy, it is somewhat incomplete to understand in detail small businesses (Rizzo & Fulford, 2012).

Structural flexibility is a dynamic capacity that allows the firm to reconfigure its structural conditions (Sharma et al.,  2010) and its decision and communication processes (Volberda, 1999) to evolutionarily adjust the organization to the particular environmental changes (Gaspary et al., 2020).

Achieving structural flexibility is also a challenging objective in most companies (Yousaf & Majid, 2018). Examples of structural flexibility include the formation of multifunctional teams, alterations in control systems, and the interchangeability of positions (Sharma et al., 2010; Volberda, 1996). Structural flexibility encompasses several structural elements: one of them is organizational design (Batra, 2006), which refers to the way work is divided and assigned among different positions, it can follow a mechanical or organic model (Sipayung et al., 2021). Another element is decision making by top managers, which can be centralized or decentralized (Batra, 2006). The level of formalization is also part of structural flexibility, and refers to the degree to which rules, policies, and procedures govern decision making and labor relations (Marín-Idárraga & González, 2021). All these elements work within a configuration hierarchy and must fit together to support the firm’s strategic planning (Burton et al., 2017; Sipayung et al., 2021). According to contingency theory, this configuration must be based on the organization’s value system and may move depending upon the contingencies of its environment (Batra, 2006; Gaspary et al., 2020).

Some studies affirm that in traditional organizations characterized by mechanistic structures with many hierarchical levels, centralized decision making and the extensive use of formal rules and procedures, structural flexibility is inhibited and they may have difficulty responding to changing environments (Gaspary et al., 2020; Sipayung et al., 2021). It seems that the concept of formalization is associated with rigidity or strictness, and can prevent a company from developing its creativity and flexibility (Gaspary et al., 2020; Sopelana et al., 2014). Top-down organizational structures are more common in large companies operating in stable environments that demand control and predictability (Mosca et al., 2021). In this sense, it is recommended that if companies want to foster their flexibility they look for a more organic model in their organizational structure, characterized by less hierarchical levels, decentralized decision-making processes, and fewer rules and formal procedures (Mosca et al., 2021; Sipayung et al., 2021). A reduction on hierarchical levels will foster an easier exchange of knowledge and problem-solving (Gaspary et al., 2020). In fact, there is a recent trend toward flat, bottom-up, and decentralized organizational structures (Mosca et al., 2021) such as a holacracy—an organizational framework that eradicates conventional hierarchies to better respond to dynamic environments (Ackermann et al., 2021). Some new ventures (e.g., GitHub (Burton et al., 2017); SMEs (e.g., Zappos (Ackermann et al., 2021); well-established firms (e.g., Mercedes-Benz.io GmbH (Ackermann et al., 2021); and firms at the beginning of their life cycle (Mosca et al., 2021) have jumped on this new wave.

However, it is important to know that some of these companies that have tried to lead organizations without bosses have not succeeded and have returned to traditional hierarchical schemes (Burton et al., 2017). To this respect, Burton et al. (2017) suggests that the effectiveness of non-hierarchical forms might not apply to all types of companies, as hierarchy continues to be essential for some of them. A better comprehension of how organizational design and decision-making processes are performed in today's companies, is still desired (Mosca et al., 2021).

Nevertheless, there is another research stream that claims flexibility and formalization are not antagonistic concepts. The “formalized flexibility” can be achieved by applying formal rules without losing flexibility altogether (Mattes, 2014). In this sense, top-down organizational structures and bottom-up organizational structures can coexist simultaneously, although it could be challenging (Mosca et al., 2021). Structural flexibility can be developed by creating interconnected networks of relationships, although they do not necessarily reflect a company’s hierarchical organization (Fioretti, 2012). For this purpose, internal communication is a fundamental pillar that provides a synergistic platform (Majid et al., 2019) for the exchange of knowledge, experiences, perspectives, and ideas (Bamel et al., 2013; Gaspary et al., 2020). Working in multifunctional teams in a work environment rich in collaboration and communication (Gaspary et al., 2020) generates continuous learning that favors a cultural change in people's mindset, essential to support both innovation and flexibility in organizations (Sushil, 2017). On the other hand, lack of communication affects employee morale and performance, leads to prejudice, excessive workloads, or duplication of duties, hindering the prompt response of the company to environmental threats or opportunities (Bamel et al., 2013).

Decision making in organizations can be centralized or decentralized. In companies with a high degree of centralization, decisions are made by the highest level of the hierarchy and their authorization is required for implementation (Castillo, 2006). In decentralized firms, decision making does not necessarily depend on a single person, but is shared with more people so that resolutions can be reached through collective consultation (Bamel et al., 2013). Companies with greater flexibility in decision making can maintain multiple alternatives and quickly modify their decisions to cope with changing environments (Kandemir & Acur, 2012). Some authors point out that centralizing decision making enables flexibility, especially in turbulent environments (Ackermann et al., 2021; Hatum & Pettigrew, 2004), while others note that involving a greater number of employees in decision making contributes to flexibility (Gaspary et al., 2020; Herhausen et al., 2021; Mosca et al., 2021), even during periods of uncertainty (Kapucu & Garayev, 2011). The sharing of decision making requires open communication, and a diverse set of knowledge and experience from teamwork members (Bamel et al., 2013). In MSMEs, the decision-making process is usually centralized in the founder (Angeles et al., 2019) unlike large companies, where this process is generally distributed across different departments (Teece, 2016). In this sense, the role of the founder has a significant impact on the development of MSMEs, since he provides the company with a strong value-based identity that enables and motivates change (Hatum & Pettigrew, 2004).

### Organizational Life Cycle

The seminal idea of OLC theory equates the growth of the company with the life cycle of a person, plant, or animal (Adizes, 1979; Jirásek & Bílek, 2018; Mosca et al., 2021). OLC is the firm development process from birth to demise and consists of individual stages formed by distinguishable patterns of change (Jirásek & Bílek, 2018). OLC theory was introduced in 1959 and has continued to be developed to the present day thanks to their holistic and comprehensive approach, which states that both the internal factors (strategic and managerial decisions) and external conditions (market and competitive pressures) trigger the company development (Mosca et al., 2021).

OLC theory informs that as companies progress through the stages, their structural configurations and strategic priorities vary significantly (Wang et al., 2020). Youth or organizational aging may be related, but not determined by the chronological age of the company (Adizes et al., 2017) or its size (Mosca et al., 2021). In this sense, the organizational development should be measured by other factors. It seems that a decrease in flexibility (Adizes, 2004), adaptability (Mosca et al., 2021), an increase in formalism (Adizes, 1979; Jirásek & Bílek, 2018), and the market growth rate (Jirásek & Bílek, 2018) are important factors to distinguish growth, development, and aging in organizations.

Although OLC theory is highly valued in changing environments and has given rise to multiple models and approaches (Jirásek & Bílek, 2018; Mosca et al., 2021; Tam & Gray, 2016a, 2016b), it does not address organizational complexity (Mosca et al., 2021). For instance, the outstanding and detailed model of Adizes (1979) provides the various sub-stages of the OLC (Wang et al., 2020) and analyzes relevant issues of organizational development, showing that the decline of the company is mainly due to the reduction of the flexibility and an overemphasis on bureaucracy (Mosca et al., 2021). However, it does not define what type of flexibility or what specific flexibility factors tend to decrease over time. In general, OLC models provide limited details on the structural and strategic characteristics of the organization at different stages. This is relevant, because capturing the multidimensionality of the relationships that link organizational elements such as structure, strategy, and environmental dimensions is necessary to build more robust theoretical business models (Soda & Furnari, 2012).

Although there is no consensus on the number of OLC stages, three common periods or stages can be clearly identified in several life cycle models: 1) founding/growth, 2) maturity/revival, and 3) decline/demise. Once a company has been legally “born,” the early phase represents a brief period of struggle for survival known as founding, conception, or infancy (Adizes, 1979; Jirásek & Bílek, 2018; Kazanjian, 1988). Having overcome this brief period through the creation of a distinguished competitive advantage and the guarantee of working capital, a stage of rapid growth follows in which the company is expected to increase in terms of size and revenues (Greiner, 1998; Hanks et al., 1994; Yi et al., 2021). Companies in these early stages may have an individualistic and entrepreneurial management style and involve frequent and informal communication between employees (Greiner, 1998). There is a family environment with limited and uninteresting hierarchy (Verma & Kumar, 2021) that allows flexibility and speed in decision making (Adizes, 1979; Broekaert et al., 2016). Regarding their relationships with other stakeholders, these organizations can be reactive or defensive (Jirásek & Bílek, 2018). Considering the preceding literature review, different characteristics of flexibility might appear throughout the MSME lifecycle. We expect strategic and structural flexibility dimensions may help identify an OLC stage. Then, our first hypothesis posits that a more flexible firm is able to stay young.

#### Hypothesis 1

Young organizations present more flexible characteristics than mature organizations.

In the following phase, also called the maturity, stability, or revival stage (Jirásek & Bílek, 2018; Kazanjian, 1988; Lester et al., 2008), profits and cash are maximized. Companies tend to lose the momentum and creativity of earlier stages, but can still be revitalized through product and market innovation (Yi et al., 2021). As organizations develop, their structure and processes gradually become formal while centralization decreases (Hanks et al., 1994). These changes are often determined by pressure and tactical factors rather than by strategy (Adizes, 1979). Companies in this stage already have a professional team of managers and a greater formalization of programs, policies, and controls (Hanks et al., 1994; Yi et al., 2021). They have clear priorities, good decision-making abilities, and well integration and communication with their stakeholders (Verma & Kumar, 2021). Management can be separated from ownership, although this process is more typical of large organizations than of MSMEs (Jirásek & Bílek, 2018).

In the final phase, also identified as the decline or demise (Hanks et al., 1994; Jirásek & Bílek, 2018; Lester et al., 2008), companies begin to collapse and lose creativity (Yi et al., 2021). Managers work hard to maintain order, while employees are primarily concerned with resolving their personal conflicts and criticizing others (Verma & Kumar, 2021). Communication is poor, power is centralized (Mintzberg, 1984), and the structure is unsound and bureaucratic (Adizes, 2004; Verma & Kumar, 2021). No strategy can flourish in this stage (Lester et al., 2008). The firm is not able to generate the resources it needs to sustain itself and its death is imminent (Verma & Kumar, 2021). However, some authors do not consider this stage to be necessarily the last in the life of the company, since there is still the possibility that a successful rebirth may occur (Jirásek & Bílek, 2018). According to the last two stages (maturity and decline), we wonder if the least flexible companies are those that are near the end of their life cycle; therefore, we propose the second hypothesis:

#### Hypothesis 2

Mature organizations present more flexible characteristics than declining organizations.

**Fig. 1:**
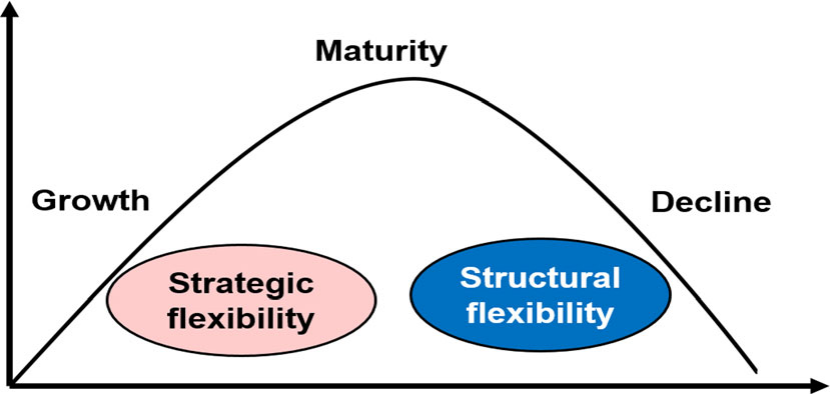
Theoretical model of strategic, structural flexibility, and OLC

Figure 1 illustrates the theoretical model aiming to relate strategic and structural flexibility to the main OLC stages in MSMEs.

## Methodology

### Sample

We focus our data collection efforts on Mexican MSMEs engaged in commercial, industrial, and service activities located in Mexico City and its metropolitan area, for the following two reasons. First, these three activities represent the 98.5% of the private sector companies and contribute to the 91.5% of the total employed personnel (INEGI, 2014, 2019). Second, the central region of the country concentrates the highest percentages of commercial and service MSMEs companies. Particularly, in Mexico City and the State of Mexico operate 24.2% of these commercial companies, 21.3% of service business, 16.3% of manufacturing companies of the country. This highlights the importance of commercial and service activities in metropolitan areas (INEGI, 2014, 2019). The data were gathered during 2014 and 2015.

Following the criterion of the Mexican Secretary of Economic Affairs (Secretaría de Economía, 2009), this research classifies MSMEs according to their number of employees: micro-enterprises 1–10; small enterprises 11–50; medium enterprises 51–250. We applied the following criteria to select the sample of Mexican firms: (a) they must be active and operating at the time of the survey; (b) be privately owned enterprises; (c) MSMEs dedicated to commercial, service, or industrial activities. Since the MSME criterion was required of all companies, we did not consider it necessary to limit the selection of firms by type of industry. Companies with more than 250 employees, state-owned organizations, and firms dedicated to other economic activities (e.g., agriculture, animal husbandry, construction, electricity, fishing, forestry, mining, gas and water supply, storage and transportation) are considerably different (INEGI, 2019) and are not part of this study.

### Data Collection

The unit of analysis is the firm level. For that reason, we follow a key informant approach to obtain the required information from the most knowledgeable person about our research topic (Kumar et al., 1993). Even though this approach can lead to potential bias (Kumar et al., 1993), it is considered appropriate in strategic management research, where there are only a few qualified respondents in the organization (Huber & Power, 1985; McGee & Sawyerr, 2003). Owner–managers are key informants as they play an important role in shaping organizational variables and receive information from a variety of departments (Huber & Power, 1985).

To mitigate the risk of common method bias (CMB), we devoted much attention to ex-ante procedural strategies (Podsakoff et al., 2003). In applied and managerial settings like ours, the use of research design solutions before data collection provides higher quality solution to CMB concerns (Jordan & Troth, 2020). For instance, to increase the probability of response accuracy, we gave a set of instructions to respondents letting them know the purpose of the research and how the information would be used (Hair et al., 2019). We also highlighted the absence of correct or incorrect answers to reduce social desirability bias (Podsakoff et al., 2012). As it was not possible to measure the predictor and criterion variables in different times or locations, we attempted to minimize the evaluation apprehension by guaranteeing response anonymity (Podsakoff et al., 2003; Tehseen et al., 2017). Additionally, we promised feedback to anyone willing to provide their email address at the end of the survey to encourage greater accuracy (Jordan & Troth, 2020). Furthermore, we also attempted to reduce CMB in the questionnaire design; we removed similar scale properties by altering the anchor labels of the response formats that measure the main constructs, as recommended by Jordan and Troth (2020) and Podsakoff et al. (2012). For instance, strategic flexibility responses were obtained on a 5-point Likert-type scale from 1 (“Strongly Disagree”) to 5 (“Strongly Agree”). Structural flexibility responses were on a 5-point scale from 1 (“Never”) to 5 (“Always”). OLC stage was determined using a multiple-choice scale. This questionnaire is available upon request from the corresponding author of this study.

After the data collection (ex-post), we conducted Harman’s one-factor test (Podsakoff et al., 2003) to reduce concerns over the impact of CMB on our results. We entered all items of the two independent variables and one dependent variable into an exploratory factor analysis, with principal axis factoring as extraction method in SPSS 24. The generated PCA output manifested 14 distinct factors accounting 63.4% of the total variance. The first unrotated factor concentrated only 17.4% of the variance in data. Thus, the two underlying assumptions did not occur (i.e., no single factor emerged and the first factor did not capture most of the variance). Therefore, these results suggested that CMB is not a pervasive issue in our study (Fuller et al., 2016; Podsakoff et al., 2003; Tehseen et al., 2017).

### Survey Design and Administration

It is important to mention that, to identify the OLC stage, it is necessary to obtain specific data on organizational culture, structure, strategies, business results, internal conflicts, management styles, plans, and compensation. Such data are considered sources of competitive advantage and certain companies are reluctant to share them with strangers with whom they have no relationship, need or obligation (Adizes et al., 2017). This is the case particularly in Mexico, where companies have been affected by organized crime and persistent violent insecurity in the last decades (Schultze-Kraft et al., 2018), and owner–managers might be fearful and unwilling to share such information.

For this reason, we decided to direct the study’s data collection efforts toward suitable and sufficient MSMEs through convenience sampling. Similar studies have analyzed the characteristics and development of organizations throughout their life cycle using a non-probability quota sample (Adizes et al., 2017; Ochoa Jiménez et al., 2021; Tam & Gray, 2016b). We acknowledge the criticism of this technique for its limited generalization (Jager et al., 2017). However, simply categorizing convenience samples as good or bad, unnecessarily slows the advancement of knowledge (Landers & Behrend, 2015). In certain circumstances, convenience samples do not damage the external validity of research studies (Landers & Behrend, 2015). For instance, homogeneous convenience samples provide more accurate population estimates from a more circumscribed population (Jager et al., 2017). As the sample size increases, the statistical power of the convenience sample also increases (Etikan et al., 2016). In studies that seek to better support the development of theoretical frameworks (Locke, 2001), random sampling is neither necessary nor preferable (Eisenhardt, 1989). Convenience sampling allows easy accessibility, availability, geographic proximity at a given time, and willingness to participate (Etikan et al., 2016). All these reasons made sense for the researchers at the time of the fieldwork.

The research questionnaire was personally delivered to the owner–managers of the company with the support of a group of trained assistants. This method is considered more reliable than surveys sent by mail as the latter is extremely difficult to carry out in Mexico, especially among micro-enterprises, due to their limited internet connectivity (only 9% of industrial companies, 13% of commercial companies, and 25% of service companies have this access) (INEGI, 2014).

A total of 300 responses were obtained from the owner, who in many cases was the MSME manager. The survey responses were thoroughly examined, and we have discarded 43 unusable responses, which contained missing information. Our final Mexican sample included 257 valid questionnaires. We provide a profile of the respondents in Table 3.

**Table 3:** Profile of the Mexican MSMEs sample

	n = 257	Percentage
*Firm size (number of employees)*		
1–10	151	59%
11–50	82	32%
51–250	24	9%
*Firm age*		
Less than 1 year	21	8%
Between 1 and 2 years	47	18%
Between 2 and 5 years	40	16%
More than 5 years	149	58%
*Firm activity*		
Industry	20	8%
Commerce	106	41%
Service	131	51%
*Firm property*		
Family	149	58%
Non-family	106	42%

### Instruments and Data Analysis

To measure strategic flexibility, we rely on the reasoning of Volberda (1999), who proposes two levels: internal (or reactive) and external (or proactive). The items were adapted from the validated scale of Tamayo-Torres et al. (2010) that had been used in similar studies (Verdú-Jover et al., 2006). The scale is made up of 8 Likert-type items. Although the scale had already been validated, it was adapted to our context, so the assessment of a measurement model for strategic flexibility was performed following a confirmatory factor analysis (CFA) using EQS 6.1. Structural flexibility was measured using the scale validated by Castillo (2006), who applied this scale in organizations in the Latin American context. This instrument contains 23 five-point Likert-type questions. To identify the stage of the MSME life cycle, we apply the Adizes model (2004, 1979). Following the parsimony criterion, this study uses three representative stages of this model: growth, maturity, and decline. This practice has also been adopted in similar studies (Masurel & van Montfort, 2006; Moy & Luk, 2003; Rutherford et al., 2003; Tam & Gray, 2016b). For the analysis of the flexibility nuances during the main stages of the OLC, we combined three multivariate techniques: exploratory factor analysis (EFA), using principal component analysis with Varimax rotation, two-step clustering, and decision tree analysis. All these techniques were performed using SPSS 24.

## Results

To identify the latent variables of flexibility, items related to structural and strategic flexibility were factor analyzed. EFA analysis revealed two factors of strategic flexibility and five factors of structural flexibility. The factors were retained according to the following criteria: eigenvalues greater than or equal to 1, factors above the break in the scree plot, and a minimum of 0.50 for factor loadings (Mathijssen et al., 2017). The detailed EFA results are presented in Tables 4 and 5, respectively. The first factor of strategic flexibility was “reactive strategic flexibility,” which included items relating to measures taken by the company to adapt to its environment. The second factor was “proactive strategic flexibility,” which included actions carried out by the organization to influence its external environment.

**Table 4:** Factor analysis results for strategic flexibility

Item	Variable	Reactive Flexibility	Proactive flexibility
T2	Variety of strategic measures to deal with change	.82	
T1	Quick strategy reformulation when required by market conditions	.82	
T4	Quick delivery of products without high costs	.69	
T3	Technology allows a large number of operations	.63	
T7	Influence on political actions		.82
T6	Control and make difficult for new competitors to enter		.72
T8	Each year we make many changes in our products		.64
T5	Advertising campaigns that influence consumers behavior		.61
	Eigenvalues	2.77	1.58
	% of explained variance	34.67	19.77
	Cronbach’s alpha	.74	.66

Alpha total = 0.72; total variance = 54.44; KMO = 0.723; Bartlett spherical test = 426.731; significance = 0.000

**Table 5:** Factor analysis results for structural flexibility

Item	Variable	Formalization	Management team	Communication	Organizational design	Decision making
S1	Policies and procedures	.60				
S3	Defined reports	.60				
S5	Rewards and incentives	.59				
S6	Expenses are planned	.66				
S7	Plans tend to be formal	.60				
S8	Operational budgets	.64				
S9	Communication documented	.60				
S14	Initiative and risk taking		.60			
S15	Creativity of the group		.64			
S16	Team of specialists		.72			
S17	Different vision of the teams		.55			
S2	Job descriptions		.51			
S22	Specialists decisions		.66			
S4	Organizational chart		.60			
S10	Coordination of tasks			.69		
S11	Communication-initiative			.78		
S12	Informal communication			.61		
S13	Decisions communicated			.60		
S18	Flat organizational structure				.78	
S19	Job security				.59	
S20	Decision making in all levels				.62	
S23	Decisions can be rethought				.54	
S21	Decision making relies on a single individual					.85
	Eigenvalues	6.57	2.69	1.27	1.23	1.06
	% of explained variance	28.55	11.69	5.53	5.33	4.59
	Cronbach’s alpha	.80	.82	.71	.65	

Alpha total = 0.87; total variance = 55.69; KMO = 0.878; Bartlett spherical test = 1926.24; significance = 0.000

We used maximum-likelihood confirmatory factor analysis to evaluate the construct validity and reliability of strategic flexibility measures. All item loadings on both constructs were statistically significant; thus, convergent validity was supported. Composite reliability estimates were 0.7 and 0.8, respectively, while discriminant validity revealed that the model has different constructs for the two factors analyzed. Strategic flexibility indices indicate a good fit for the model: normed fit index (NFI) = 0.92, incremental fit index = 0.96, comparative fit index (CFI) = 0.96, and a root mean square error of approximation (RMSEA) = 0.059, despite the significance of the Chi-square value (x^2^ = 33.23, p value = 0.01564). In sum, these tests confirm the validity and reliability of the continuous variables that reflect the intended constructs.

Regarding structural flexibility, the solution identifies five factors: formalization, management team, communication, organizational design, and decision making. The last factor is composed of a single item, and the recommendation would be to disregard it (Costello & Osborne, 2005). Nevertheless, previous studies (Angeles et al., 2019) have sought to explain the decision-making factor as a freestanding item factor and concluded that it is an important determinant of the flexibility of MSMEs. For that reason, it was decided to keep this factor as part of the structural flexibility solution.

**Fig. 2:**
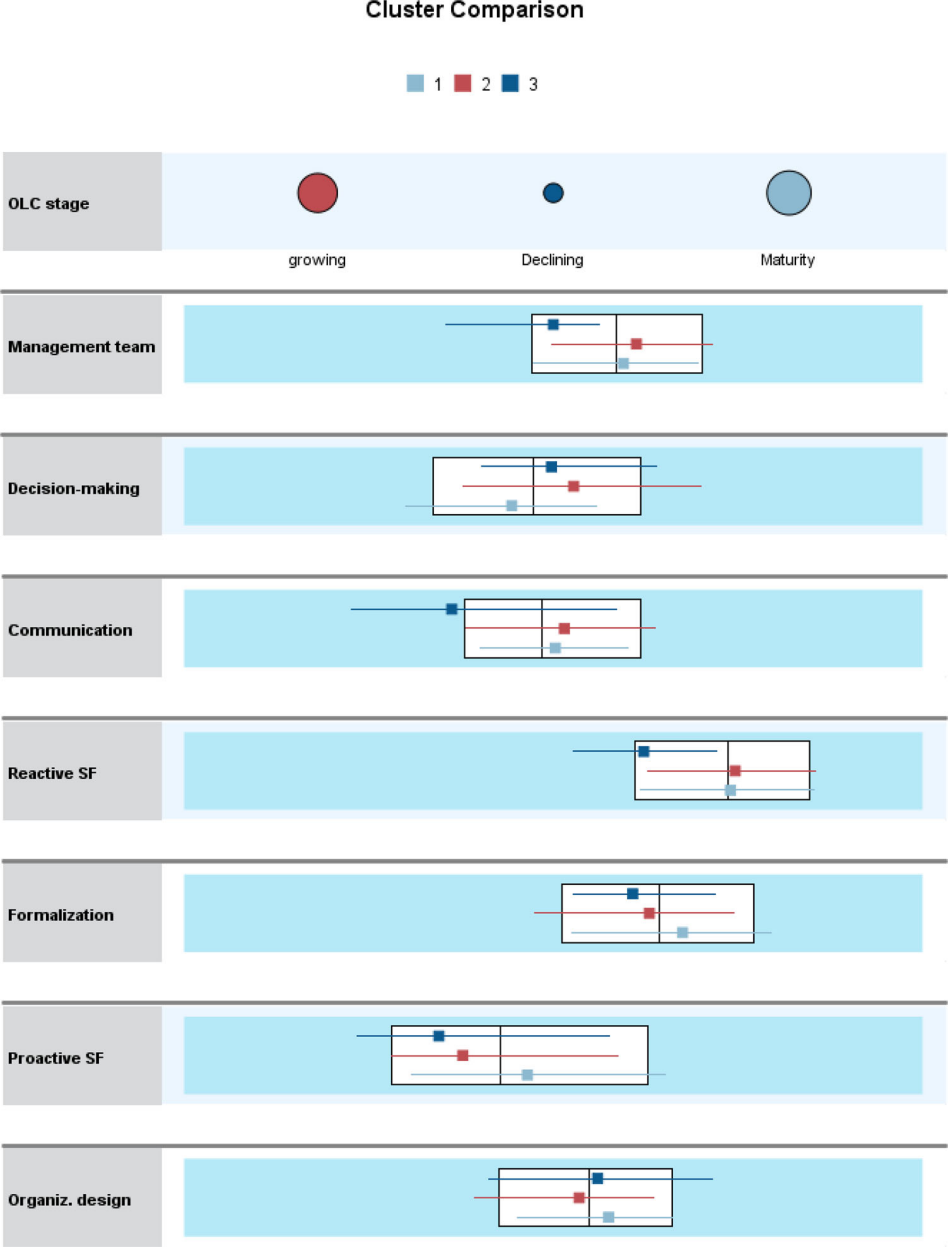
Three-cluster solution of strategic and structural flexibility in OLC stages

**Fig. 3:**
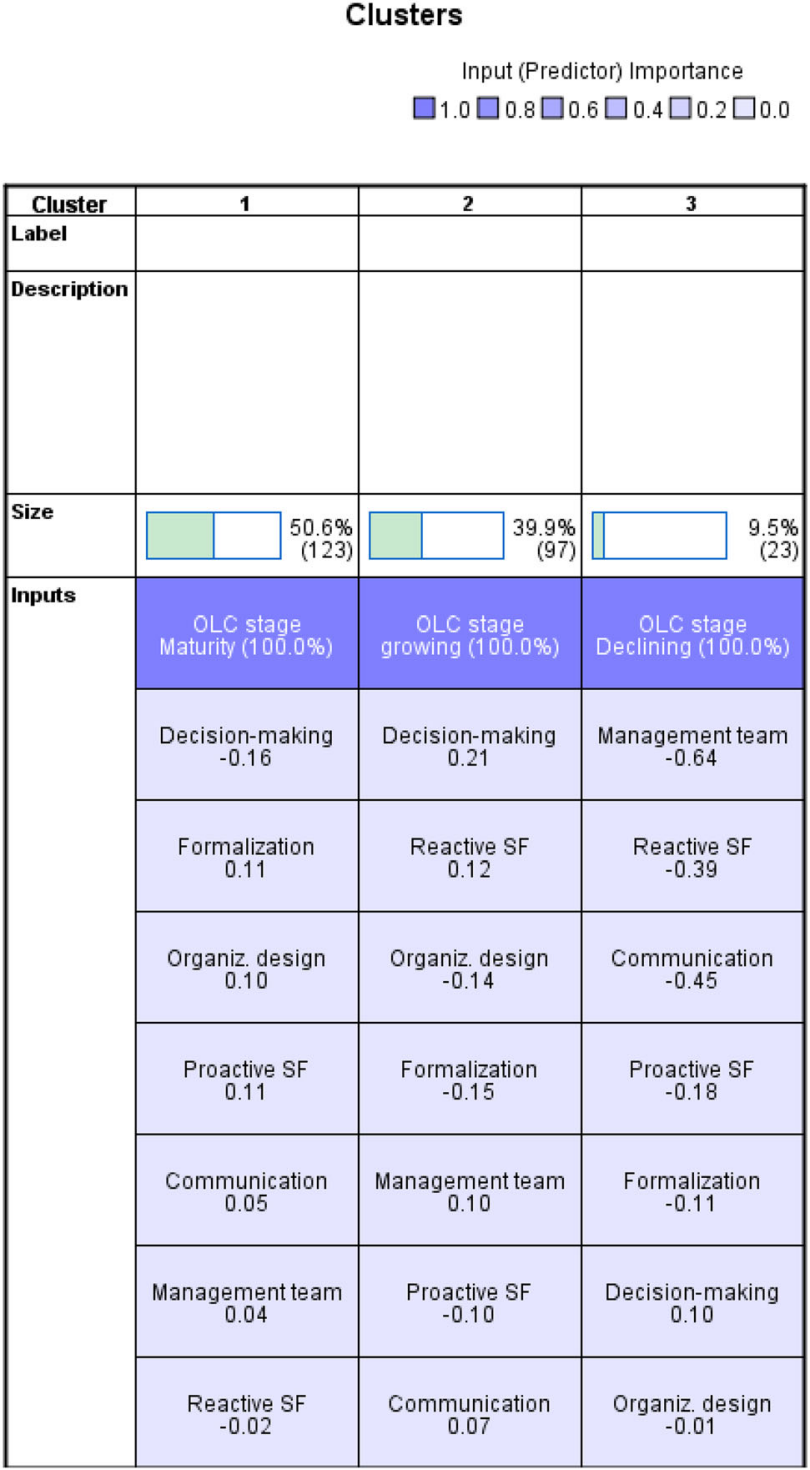
Mean values of three-cluster solution

After the EFA analysis, we carried out a two-step cluster analysis technique. Compared to other segmentation methods, this technique offers greater reliability and precision (Nurosis, 2007). It is used in social research because it helps to obtain and explain more information to improve managerial decision making (Tkaczynski, 2017). The chosen segmentation method allows us to select the number of clusters a priori. Since the objective of the analysis is to identify the characteristics of flexibility in the OLC stages, three clusters were predefined. We use the EFA results of strategic and structural flexibility as continuous variables, and OLC stage as a categorical variable. For this reason, the log-likelihood algorithm was selected as a measure to assess similarity (Tkaczynski, 2017). The silhouette value measure was 0.3, meaning that cohesion among variables of the same cluster and separation of the clusters are appropriate. Figure 2 shows the comparative cluster solution, and the mean value results are displayed in Fig. 3.

Figure 2 shows a dot plot for each stage of the categorical OLC variable. The dot size corresponds to the percentage of cases. The flexibility continuous variables are shown in a boxplot with the distribution of mean values within each group. These plots help identify the characteristics of the following clusters.

*Cluster 1. Most Flexible Firms*

This segment represents approximately 50.6 percent of the total sample, with a total of 123 firms. It presents mostly positive mean values for all dimensions of flexibility except for reactive flexibility (barely below 0) and decision making (-0.16). It encompasses firms that are in the mature stage of their OLC.

*Cluster 2. Middle-Flexible Firms*

This group represents approximately 39.9 percent of the total sample, with a total of 97 firms. It presents positive mean values in four of the seven constructs of flexibility: reactive flexibility, management team, communication, and decision making. It encompasses growing firms.

*Cluster 3. Non-flexible Firms*

This segment represents approximately 9.5 percent of the total sample, with a total of 23 firms. It presents negative mean values for all the flexibility dimensions, except one: decision making. It encompasses declining firms.

### Decision Tree

**Fig. 4:**
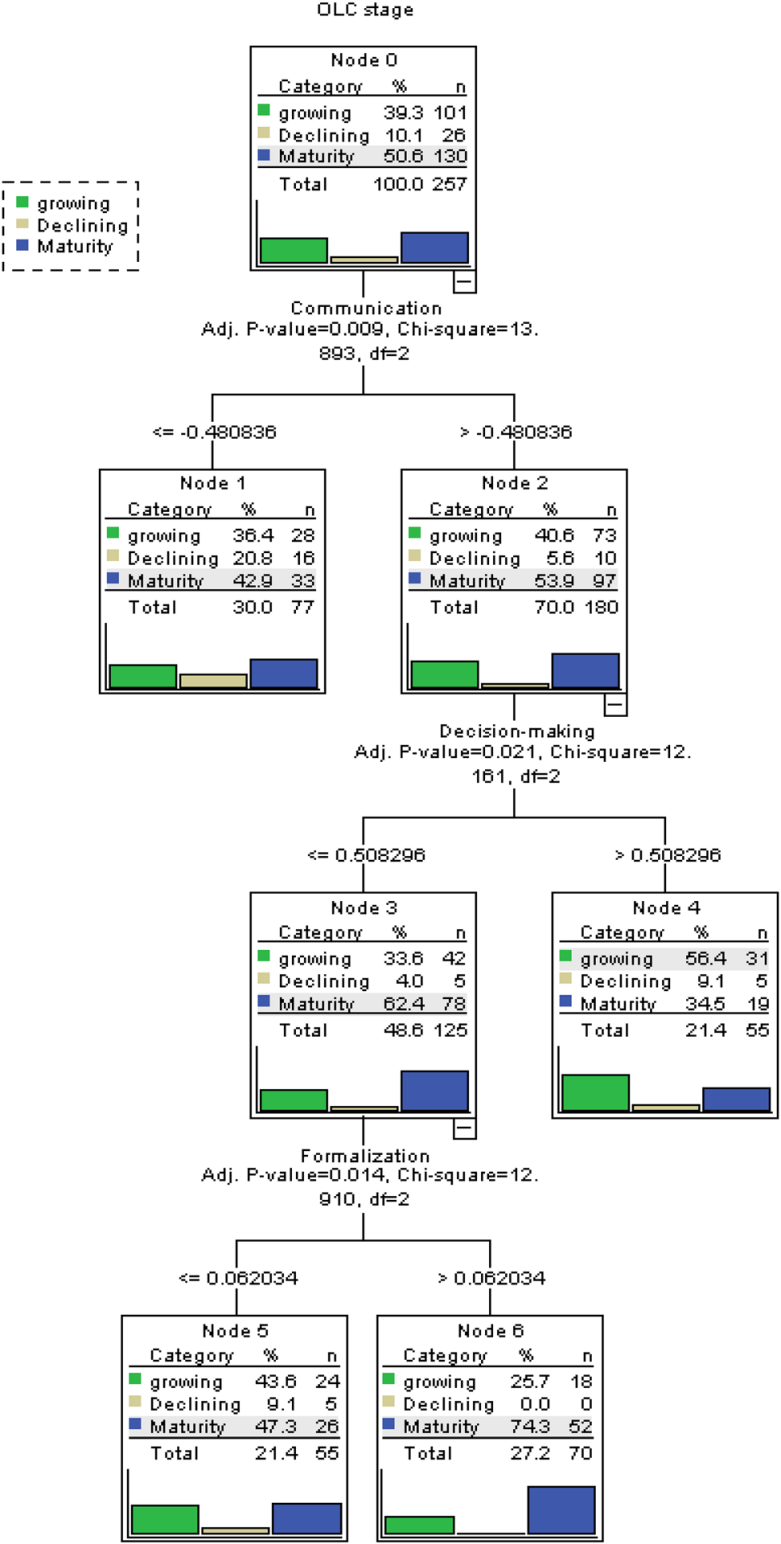
Decision tree solution

Decision tree modeling combined with cluster solution provides informative features or emerging patterns for predictive classification (Myles et al., 2004). The Chi-square automatic interaction detector (CHAID) algorithm used in this analysis, begins by finding independent variables that have a significant association with the dependent variable, the OLC stage. The first branch in a tree represents the independent variable that has the strongest association with the target variable (Thomas & Galambos, 2004). Table 6 shows the decision tree table statistics. The results of the decision tree analysis using the CHAID algorithm are shown in Fig. 4. We observe that three of the seven variables of strategic and structural flexibility—communication, decision making, and formalization—are the ones that best help classify mature MSMEs of the sample with 85% accuracy. The first criterion for classifying a mature-stage company is a high level of communication. The next criterion is decentralized decision making. Finally, the highest level of formalization helps predict whether the company is in a stage of maturity.

**Table 6:** Decision tree table statistics

Node	Growing		Declining		Maturity		Total		Predicted category
	N	Percent	N	Percent	N	Percent	N	Percent	
0	101	39.3%	26	10.1%	130	50.6%	257	100.0%	Maturity
1	28	36.4%	16	20.8%	33	42.9%	77	30.0%	Maturity
2	73	40.6%	10	5.6%	97	53.9%	180	70.0%	Maturity
3	42	33.6%	5	4.0%	78	62.4%	125	48.6%	Maturity
4	31	56.4%	5	9.1%	19	34.5%	55	21.4%	growing
5	24	43.6%	5	9.1%	26	47.3%	55	21.4%	Maturity
6	18	25.7%	0	0.0%	52	74.3%	70	27.2%	Maturity

Parent node	Primary Independent Variable				
	Variable	Sig.^a^	Chi^2^	df	Split Values
0	Communication	.009	13.893	2	< = -.480836
0	Communication	.009	13.893	2	> -.480836
2	Decision-making	.021	12.161	2	< = .508296
2	Decision-making	.021	12.161	2	> .508296
3	Formalization	.014	12.910	2	< = .062034
3	Formalization	.014	12.910	2	> .062034

Growing Method: CHAID. Dependent Variable: OLC stage. a. Bonferroni adjusted

RF = Reactive flexibility PF = Proactive flexibility F = Formalization M = Management team C = Communication D = Decision-making O = Organizational design*Source: Own elaboration*

## Discussion

**Fig. 5:**
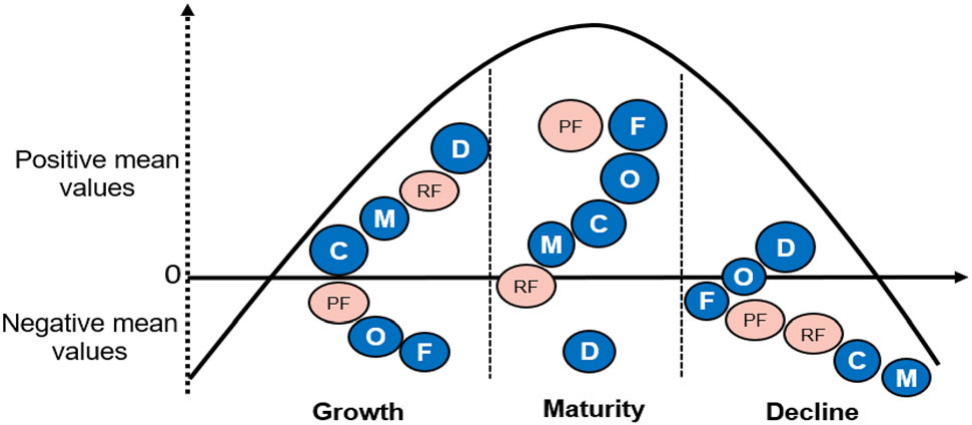
Strategic and structural flexibility dimensions throughout OLC stages

This study sheds light on unaddressed issues regarding flexibility during the life cycle of MSMEs. To better illustrate how strategic and structural flexibility help characterize and predict the growth, maturity, and decline of these organizations, we present a model in Fig. 5 with the study results explained below.

### Explanation of the Model

Mean flexibility values are shown along the OLC curve, and the circles in the upper part inside the curve reveal the positive and predominant presence of the flexibility factor at each stage. At the bottom of the curve, the circles suggest a negative and weak visibility of this type of flexibility in the indicated stage. The blue color of the circles corresponds to the structural flexibility factors, while the pink color distinguishes the strategic flexibility factors.

#### Growing Firms

Regarding strategic flexibility in MSMEs, there are two contradictory views in the literature. On the one hand, some scholars attribute this capability to large companies due to their potential availability of resources (Pauwels & Matthyssens, 2004). At the same time, they state that small businesses do not have the latitude to implement strategic flexibility because their owner–managers have little ability to understand and apply strategic tools (Woods & Joyce, 2003) as they simply do not have a strategy (Rizzo & Fulford, 2012). Some other studies defend the possibility that MSMEs are strategically flexible, appealing to their adaptability and agility (Ebben & Johnson, 2005) derived from their small and simple organizational structure (Zhang et al., 2014). These versions, however, have not delved into what type of strategic flexibility MSMEs may or may not have, and at what stage of their OLC this dynamic capability is manifested.

The results of our study help clarify this debate by showing that indeed, MSMEs do have the characteristics of being strategically flexible, but only internally or reactively, especially during the growth stage of their OLC. However, they fail to be strategically flexible in the external or proactive sense. This means that MSMEs that have already survived the start-up phase and are trying to move to a later stage of development, adapt well by modifying their processes and strategies within the company, but are unable to influence their external environment to promote or stop the changes that concern them.

Regarding structural flexibility, the growing firms in our study demonstrated this ability, particularly with respect to three factors: management team, decision making, and communication*.* This means that these companies have a diverse work team in terms of their experience, knowledge, and ideas, although the final decisions are usually made by a single person (the owner or founder). However, there is open, fast, and fluid communication among their members, which encourages initiative and risk taking. All these characteristics give growing MSMEs the ability to quickly adjust their structure to changes in the environment. In this sense, we agree with the study of Miroshnychenko et al. (2020) who highlights the importance of internal resources/drivers in these companies to enhance its development and strategic flexibility. Nevertheless, it would be advisable for the owner–managers of growing MSMEs to connect and explore more with the external environment for more proactive strategic flexibility.

#### Mature Firms

Mature firms also reveal characteristics of being strategically flexible. The main difference from growing companies is that they are more externally flexible (or proactive), meaning that they are successful in using their market power to influence their external environment (e.g., modifying consumer habits or preventing the entry of new competitors). Similar to growing companies, mature firms also display structural flexibility characteristics stemming from their fluid communication and diverse management teams. Interestingly, mature companies present two other structural flexibility factors that growing companies do not: formalization and organizational design. Decision-making factor is not present at this stage. This means that mature companies have formalized their processes and plans. At the same time, the assignment of responsibilities, power, and decision-making procedures no longer depend on a single person, as in the previous stage. Comparing the strategic and structural flexibility factors of mature companies with those of growing firms, we reject our first hypothesis: “Young organizations present more flexible characteristics than mature ones.”

In this sense, our results seem to contradict previous studies in the literature that have indicated that it is very difficult for MSMEs in emerging countries to achieve structural flexibility due to their lack of resources and structural design mechanisms (Zhang et al., 2014). Some other authors affirm that owner–managers of these companies are not very interested in the formalization and planning aspects because they associate them with the bureaucracy of large companies, and some even consider that planning is a waste of time (Woods & Joyce, 2003). There is even a strong tendency to abolish formal and traditional hierarchies, and run businesses where employees apply their own judgment instead of following standardized rules to solve problems (Ackermann et al., 2021). This negative view of MSMEs has not specified other characteristics beyond their size as small companies. Our findings help to detail that in mature MSMEs, formalization is not synonymous with rigidity. On the contrary, these control mechanisms that appear in this stage can coexist with flat organizational designs and decentralized decision making. It should be noted that flexibility in this stage is accompanied by open communication and a heterogeneous management team. All these conditions allow mature MSMEs greater structural flexibility.

As far as the holacracy is concerned, we do not fully agree with Ackermann et al. (2021) who point out that this new trend seems appropriate for companies where the need for adaptability exceeds the need for reliability. We consider this practice is not an out-of-the-box solution to increase flexibility, especially for MSMEs going through mature OLC stages in changing development contexts. In this sense, we conceive the theoretical possibility of an intermediate configuration between the formalization and flexibility. Our results are more in line with Burton et al., (2017) and Mattes (2014) who promote the “formalized flexibility,” where formalization and flexibility do not contradict but rather complement each other. We agree with Mosca et al. (2021) that this hybrid organizational configuration could be challenging, and with Sushil (2017), that great care must be taken to improve flexibility without losing controllability, otherwise the organization may age prematurely.

Additionally, it would be advisable for the owner–managers of mature MSMEs to identify and decide on different levels of decentralization and formalization as their companies evolve, and to assess the pros and cons of carrying out these changes. In line with the ideas of Sushil (2017), we also assert that it is not enough to be a flexible company, or to keep a young company forever. There is something else that mature or growing MSMEs can do, and that is to bring their benefits to all their stakeholders. It is of little use for a company to remain flexible or mature if its ecosystem is in chaos. Environmental, political, or health crises cannot be addressed in isolation; therefore, it is necessary to collaborate and share the benefits with related actors and jointly face the unprecedented uncertainties in today’s business environment.

#### Declining Firms

The last part of our findings shows that companies in declining stages have neither reactive nor proactive strategic flexibility. This means that they have difficulties in modifying their internal processes, and they can do little or nothing to influence their environment to generate opportunities or defend themselves from threats. This is in line with Woods and Joyce (2003) who agreed that owner–managers of declining firms use few strategic tools, and do not consider this to affect their ability to run their firms strategically.

Regarding structural flexibility, declining companies showed the presence of only one factor, decision making, which means that this process still depends on a single individual. They lost (or never developed) other flexible characteristics (e.g., fluid communication, diverse management teams, formalization, and organizational design). Therefore, H2 is confirmed. “Mature organizations present more flexible characteristics than declining organizations.”

With regard to centralization in decision making, some research streams indicate that it allows flexibility because decision making is faster (Ackermann et al., 2021; Hatum & Pettigrew, 2004), while others point out that it inhibits it (Herhausen et al., 2021; Mosca et al., 2021). Our results help clarify this debate, as we note that it is precisely in the growth and declining stages, where a single individual usually makes all the decisions. The difference is that in the declining stage, decision making is not accompanied by other flexibility factors. This can become an obstacle to flexibility, especially for the aging companies. This is in line with Adizes (2004) who points out that decision making dominated by an individual can be a normal problem in growth stages, but if it occurs in more advanced stages, it can be considered an abnormal problem. We also agree with Eisenhardt (1989) who shows that centralization does not solve other problems when deciding under uncertainty.

Finally, our findings suggest that MSMEs emphasize different characteristics of flexibility throughout their life cycle. For example, in our analysis we did not find the presence of all the flexibility factors (strategic or structural) together in any stage, nor did we observe any common factor that was always present in the stages analyzed. However, our results allow us to identify three main variables that help predict and classify a mature company: high levels of communication, decentralized decision making, and progress in establishing formal processes within the firm. This supports Jirásek and Bílek (2018), who mention that formalism is one of the main factors to distinguish the stages of OLC. With these results, our study enriches the model of Adizes (1979, 2004), because we detail the different types of flexibility that can be found in the OLC stages. It also contributes to the efforts of other authors (Zhang et al., 2014) who seek to distinguish the role played by different types of flexibility in MSMEs immersed in a constantly changing environment.

## Conclusions

This research delves into the nuances of two types of flexibility, strategic and structural, and links them to the organizational life cycle of MSMEs in a developing country. It brings new knowledge to the more traditional studies that consider flexibility as a general concept and that have been carried out in large companies in developed countries. This article yields several relevant implications in two areas: theoretical and managerial.

### Theoretical Implications

Some OLC models have shown that organizations evolve regardless of their size and chronological age, and it is the decrease in flexibility and adaptability that determines organizational youth or aging. However, little attention has been given to examining the nuances of this flexibility. The main contribution of our article is to provide empirical evidence of which strategic and structural flexibility factors help characterize and predict the stages of growth, maturity, and decline of MSMEs. In this way, we extend the OLC model for MSMEs and join the efforts of various authors to move toward a more robust OLC theory.

Our results show that mature companies have more characteristics of being strategically and structurally flexible than those in stages of organizational growth or decline. Flexibility factors that help classify and predict a mature MSME include open communication, decentralized decision making, and formalization. We provide a model with these results to illuminate unaddressed issues regarding the broad term flexibility and its relationship to OLC.

### Managerial Implications

Several managerial implications derived from our study suggest that owner–managers of MSMEs need to understand better how strategic and structural flexibility affects its organizational aging. Referring to the growing MSMEs, they must connect and explore more with the external environment for greater proactive strategic flexibility, while maintaining the dominant factors of their reactive strategic flexibility to keep their companies alive and avoid premature death. Owner–managers of mature MSMEs that are challenged to achieve “flexible formalization” need to identify effective coordination mechanisms and decide on the appropriate level of decentralization and formalization, and not simply follow new trends, such as holacracy, without having the necessary elements to do so. For the overwhelmed owner–managers of MSMEs in the decline stage, they must consider that, in a complex, globally dispersed, and strictly scrutinized environment such as the current one (Mosca et al., 2021), they will not be able to manage the growth of their companies alone. They must consider other factors that ensure greater flexibility to overcome this stage, for instance, a more inclusive organizational design that contemplates a heterogeneous management team, as well as flexible formalization and communication mechanisms. For business consultants who advise MSMEs, it is relevant that they consider the importance of the relationships between strategic and structural flexibility during the different stages of the OLC, when providing advice and support on the subject of business development.

### Limitations and Future Research

Our study comes from a cross-sectional study based on key informants from Mexican MSMEs. It has several limitations that are also valuable indications for future research. For instance, despite the economic importance of MSMEs, which constitute 95% of the country’s companies, obtaining information about possible sources of competitive advantage of these companies is almost impossible without a strong connection with business owners–managers and their willingness to answer the research questionnaire (Adizes et al., 2017), even more so in a context of national insecurity. For these reasons, convenience sampling was used to obtain the data, and this is one of the weaknesses of our study. Therefore, no claims are made about its generalizability. Although we performed ex-ante strategies and ex-post statistical test (e.g., Harman’s one-factor test) to rule out potential bias concerns, this study does not address other tests to detect CMB here.

Research on MSMEs could develop additional analyses to test our results in different contexts where cultural and institutional settings may vary, using a longitudinal perspective to improve the generalizability of our results across these companies. They could also delve into how they can achieve the recommended middle ground of maintaining a formal organizational design without losing flexibility. Future OLC studies could contemplate the use of other key variables that help to determine OLC stage, such as the growth rate of the market. Additionally, studies focused on family businesses that contrast with non-family businesses could provide new insights on this topic.

Finally, as researchers in an emerging economy, we call on our colleagues to develop frameworks that allow us to understand the nuances of our contexts, since, as we noted earlier, business models from developed countries do not necessarily fit the circumstances of organizations in developing countries. If the results of this work encourage further research in this same direction, then it will have accomplished its goal.

Key Questions

1. What other flexibility factors do you consider could affect the aging of your organization?
2. How could the lessons of this article be applied to MSMEs in your context?
3. To what extent the results of this research open new paths towards a better development and growth of your company?
